# Targeting immune cell types of tumor microenvironment to overcome resistance to PD-1/PD-L1 blockade in lung cancer

**DOI:** 10.3389/fphar.2023.1132158

**Published:** 2023-02-15

**Authors:** Man Wang, Lijie Zhu, Xiaoxu Yang, Jiahui Li, Yu’e Liu, Ying Tang

**Affiliations:** ^1^ Department of Respiratory Medicine, The First Hospital of Jilin University, Changchun, Jilin, China; ^2^ Tongji University Cancer Center, Shanghai Tenth People’s Hospital of Tongji University, School of Medicine, Tongji University, Shanghai, China

**Keywords:** TME, PD-L1, PD-1, time, immunotherapy, resistance

## Abstract

Lung cancer is the common malignant tumor with the highest mortality rate. Lung cancer patients have achieved benefits from immunotherapy, including immune checkpoint inhibitors (ICIs) therapy. Unfortunately, cancer patients acquire adaptive immune resistance, leading to poor prognosis. Tumor microenvironment (TME) has been demonstrated to play a critical role in participating in acquired adaptive immune resistance. TME is associated with molecular heterogeneity of immunotherapy efficacy in lung cancer. In this article, we discuss how immune cell types of TME are correlated with immunotherapy in lung cancer. Moreover, we describe the efficacy of immunotherapy in driven gene mutations in lung cancer, including KRAS, TP53, EGFR, ALK, ROS1, KEAP1, ZFHX3, PTCH1, PAK7, UBE3A, TNF-α, NOTCH, LRP1B, FBXW7, and STK11. We also emphasize that modulation of immune cell types of TME could be a promising strategy for improving adaptive immune resistance in lung cancer.

## Introduction

Lung cancer is the common malignant tumor and displays the highest mortality rate (Chen P. et al., 2022; Choi and Mazzone, 2022). Lung cancer had 1.8 million deaths (18% of the total cancer-related deaths) worldwide in 2020 (Sung et al., 2021). Lung cancer is the most frequently occurring tumor in males and the third commonly diagnosed tumor in females. Lung cancer is the first cause of tumor death in males and the second leading cause of cancer mortality in women (Sung et al., 2021). In the United States, there are 2,36,740 new lung cancer cases and 1,30,180 lung cancer-related deaths (Siegel et al., 2022). The 5-year survival rate of lung cancer is only 10%–20% in some countries (Sung et al., 2021).

**FIGURE 1:**
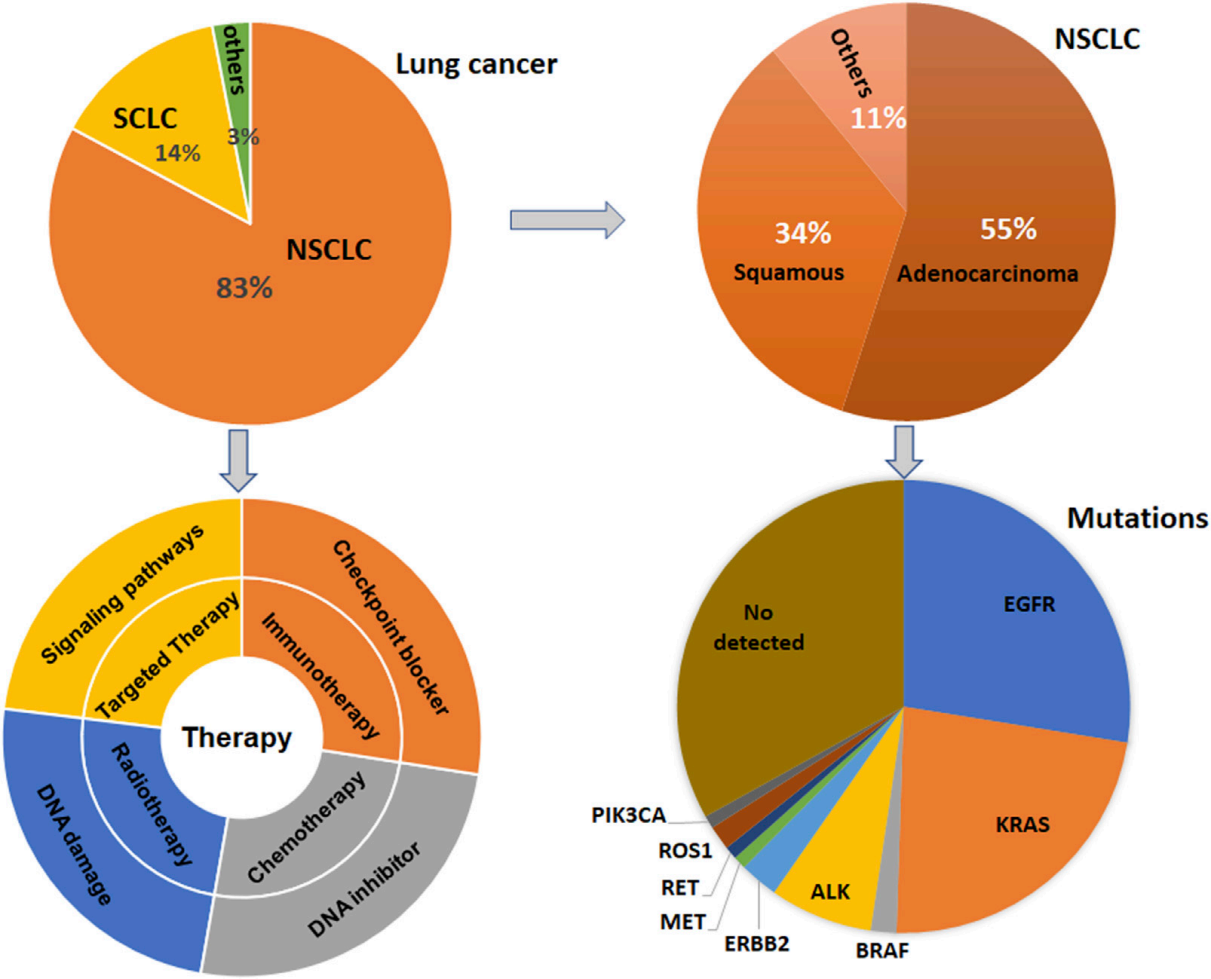
Gene Mutations and therapies are illustrated in lung cancer. Lung cancer has three-type categories, including SCLC, NSCLC, and unspecified histology. NSCLC patients have multiple gene mutations. Targeted therapy, immunotherapy, antiangiogenic therapy and combination therapy have been used in the clinic for lung cancer patients.

Lung cancer has three-type categories, including small cell lung cancer (SCLC, 14%), non-small cell lung cancer (NSCLC, 82%) and unspecified histology (3%) (Miller et al., 2022). The NSCLC includes large cell carcinoma, adenocarcinoma, and squamous cell carcinoma (Mengoli et al., 2018). The global lung cancer occurrence could be due to outdoor ambient PM2.5 and tobacco (Guo et al., 2020; Turner et al., 2020; Frazer et al., 2022). Multiple gene mutations have been found in NSCLC patient, including epidermal growth factor receptor (EGFR) (Zhao D. et al., 2022; Castaneda-Gonzalez et al., 2022), Kirsten rat sarcoma viral oncogene homolog (KRAS) (Desage et al., 2022; Garcia-Robledo et al., 2022), anaplastic lymphoma kinase (ALK) (Cognigni et al., 2022; Xiang et al., 2022), Erb-B2 Receptor Tyrosine Kinase 2 (ERBB2) (Ni and Zhang, 2021; Vathiotis et al., 2021; Yu X. et al., 2022), B-Raf proto-oncogene (BRAF) (Abdayem and Planchard, 2022; Riudavets et al., 2022; Sforza et al., 2022), phosphatidylinositol-4,5-bisphosphate 3-kinase catalytic subunit alpha (PIK3CA), AKT serine/threonine kinase 1 (AKT1), mitogen-activated protein kinase kinase 1 (MAP2K1) (Kim and Giaccone, 2018; Han et al., 2021), c-ros oncogene 1 (ROS1) (Guaitoli et al., 2021; Yu Z. Q. et al., 2022), neurotrophic tyrosine receptor kinase (NTRK) (Liu C. et al., 2022; Qin and Patel, 2022), and mesenchymal-epithelial transition factor (MET) (Pao and Girard, 2011; Olmedo et al., 2022) (Figure 1). In SCLC patients, gene mutations often include retinoblastoma (Rb), TP53, PTEN, FBXW7, VHL mutations (Cardona et al., 2019; Guan et al., 2022). In addition, targeted therapy, immunotherapy, antiangiogenic therapy and combination therapy have been used in the clinic for lung cancer patients (Luo et al., 2021; Wang et al., 2021; Guo et al., 2022) (Figure 1). NSCLC patients with KRAS mutation or EGFR often have a worse benefit from immunotherapy (Di Nicolantonio et al., 2021).

Immunotherapy has improved the therapeutic outcomes in lung cancer patients (Caliman et al., 2022; Catalano et al., 2022; Martin and Enrico, 2022; Tartarone et al., 2022; Yang et al., 2022). Immune checkpoint inhibitors (ICIs) have been used for the cancer therapy, including anti-PD-1, anti-PD-L1 and anti-CTLA-4. Anti-PD-1 drugs in NSCLC have cemiplimab, pembrolizumab, and nivolumab (Bote et al., 2022; Mussafi et al., 2022). The anti-PD-L1 monoclonal antibodies have durvalumab and atezolizumab in NSCLC. Anti-CTLA-4 (ipilimumab) is also be used in lung cancer because of CTLA-4 as a checkpoint on lymphocytes (Ackermann et al., 2019; Dawe et al., 2020; Peng et al., 2022). However, PD-1/PD-L1 monoclonal antibodies and anti-CTLA-3 treatment obtain a good response in a subgroup of lung cancer patients. Moreover, adaptive immune resistance is observed in lung cancer patients and attenuated the immunotherapeutic benefits (Gkountakos et al., 2021; Gemelli et al., 2022). Furthermore, immunotherapy often causes side-effects, such as endocrinopathy, colitis, pneumonitis, nephritis in lung cancer patients (Bredin and Naidoo, 2022; Hao et al., 2022).

Tumor microenvironment (TME) is a unique environment and composed of many other types of cells, such as stromal, endothelial and immune cells, which has shown to participate in tumor development, initiation and progression as well as metastasis (Eulberg et al., 2022; Nallasamy et al., 2022). The TME cellular components consist of MDSCs, Treg cells, M1 macrophages, M2 macrophages, cytotoxic CD8^+^ T cells and NK cells (Gajewski et al., 2013; Cao et al., 2022). It has been known that tumor cells inhibit the anticancer functions of TME and promote pro-tumorigenic functions of TME (Faraj et al., 2022; Tiwari et al., 2022). Tumor immune microenvironment (TIME) consists of tumor cells, different cell types of the immune system and their interactions in the TME niche (Binnewies et al., 2018).

In this review article, we described the association between immune cell types in TME and immunotherapy in lung cancer. Moreover, we discussed the efficacy of immunotherapy in driven gene mutations in lung cancer, including KRAS, TP53, EGFR, ALK, ROS1, KEAP1, ZFHX3, PTCH1, and STK11. Furthermore, we concluded that targeting TME could be helpful to overcome resistance to PD-1/PD-L1 blockade in lung cancer.

### Immunotherapy, driven gene mutations, and TME

TME has been identified to take part in tumorigenesis and cancer metastasis. Several adverse conditions in TME, such as acidity, hypoxia, and nutrient restriction, have been unraveled to affect the responses of immunotherapy (Li and Qiao, 2022). Moreover, TME governs immune cell functions *via* regulation of immune cells activation (Ahluwalia et al., 2021; Genova et al., 2021). In this section, the relationship of TME, driven gene mutations and immunotherapy will be summarized. Evidence dissects that immune therapy benefit is associated with driven gene mutations and smoking status in NSCLC patients. These driven genes include EGFR, KRAS, ALK, and BRAF (Skoulidis and Heymach, 2019). One study identified that the most frequently mutated genes included TP53, KRAS, ERBB2, SMAD4, ERBB4, EGFR, BRAF, and MET (Cinausero et al., 2019). In the following sections, we will describe how these driven gene mutations modulate the TIME and affect the anti-PD-1/PD-L1 therapy in NSCLC patients (Table 1). We highlight that the efficacy of immunotherapy is modulated by these key gene mutations in NSCLC patients.

**TABLE 1:** The association of gene mutations and TME, immunotherapy in lung cancer.

Gene mutation	TME	Mechanism	Therapy	Ref
KRAS-G12D	Reduction of CD8^+^ TILs and an immunosuppressive TIME	Targeted P70S6K/PI3K/AKT, decreased CXCL10/CXCL11, inhibition of HMGA2	Resistance to anti-PD-1/PD-L1 therapy	Liu F. et al. (2022)
EGFR	ILT4 increases TAMs recruitment and M2-like polarization, impairing proliferation and cytotoxicity of T cells	Targeting ERK and AKT pathways	Inhibition of ILT4 increased the efficacy of immunotherapy	Chen K. et al. (2021)
ALK	Low proportion of PD-L1+/CD8+, activated memory CD4^+^ T cells	Targeting PD-L1 pathway	Shows a poor response to ICIs	Liu et al. (2018)
ROS1	TME is active and plasma inflammatory factors is upregulated	High expression level of PD-L1 expression	Not correlated with therapy response	Lee et al. (2019), Yue et al. (2021), Bahnassy et al. (2022)
PTCH1	Associates with CD8^+^ TILs density	High PD-L1 expression	Associated with survival	Cheng P. et al. (2022)
TP53	Enriches IFN-γ signatures and TME composition; promotes suppressor immune cells, M2 Macrophage and Neutrophils	Associates with PD-L1 expression	Associates with response to ICIs treatment	Assoun et al. (2019), Sun et al. (2020), Wang S. et al. (2022)
ZFHX3	Correlates with TILs	Correlates with immune-related gene expression	Longer survival of NSCLC patients after ICIs treatment	Zhang L. et al. (2021)
PAK7	Associated with TMB, neoantigen load, copy number variation, CD8^+^ TILs	DNA damage response-related pathways	Prediction of the immunotherapy efficacy	Zeng et al. (2022)
UBE3A	Higher TILs	High expression of immune checkpoint biomarkers	Promotes the immunotherapy efficacy	Zhang N. et al. (2022)
TNF-alpha	Related with TMB, infiltrating immune cells, neoantigen load	DNA damage response signaling	Associates with immunotherapy	Lin et al. (2021)

### The efficacy of immunotherapy in KRAS-mutant NSCLC

KRAS oncogenic pathway affected TME *via* modulation of cancer-associated fibroblasts and immune cells (Dias Carvalho et al., 2018; Ceddia et al., 2022). KRAS-mutant cancer cells govern immune responses through regulation of immune cell recruitment, activation, and differentiation, leading to enhancement of protumorigenic ability and promotion of tumor cell evasion (Dias Carvalho et al., 2018). KRAS pathway controls populations of myeloid cells, T cells, fibroblasts, endothelial cells, ECM composition. In KRAS-mutant lung cancer patients, M2 macrophages, MDSCs, CD4+FoxP3+ Treg cells, IL-17-producing T helper cells displayed a pro-tumorigenic TME (Cullis et al., 2018). Studies have shown that the efficacy of anti-PD-1 and anti-PD-L1 immunotherapy was associated with promotion of immunogenicity and an inflammatory TME (Liu et al., 2020; Ceddia et al., 2022). Kirsten rat sarcoma viral oncogene homolog (KRAS) mutations are linked to superior patient response to PD-1/PD-L1 inhibitors. KRAS mutations are associated with treatment efficacy and prognosis in NSCLC (Wood et al., 2016; Ferrer et al., 2018; Roman et al., 2018). Targeting KRAS variant has be shown to have potential treatment applications in NSCLC (Ricciuti et al., 2016; Uras et al., 2020; Li J. X. et al., 2022). Notably, KRAS mutations are linked to immune therapy resistance in NSCLC patients (Kim et al., 2017; Adderley et al., 2019).

In clinic study, NSCLC patients with KRAS mutation obtained treatment benefit from immunotherapy *via* anti-PD-1 and anti-PD-L1 approaches (Liu et al., 2020). Interestingly, suppression of PD-L1 in combination with docetaxel failed to enhance an anti-tumor response in a KRAS-mutant lung adenocarcinoma mouse model (Liu et al., 2020). This study indicated that the combination of immunotherapy and chemotherapy need to be revaluated in NSCLC patients with KRAS mutations (Liu et al., 2020). Moreover, evidence has demonstrated resistance to ICIs in NSCLC with KRAS mutation *via* modulation of tumor metabolism and TME functions (Li W. et al., 2022). KRAS-G12D mutation induced immune suppression and caused the resistance to anti-PD-1/PD-L1 therapy in NSCLC. KRAS-G12D point mutation was negatively associated with PD-L1 expression level and CXCL10/CXCL11, resulting in a reduction of CD8^+^ TILs and an immunosuppressive TIME (Liu F. et al., 2022). KRAS-G12D mutation reduced PD-L1 expression through P70S6K/PI3K/AKT pathway and decreased CXCL10/CXCL11 expression *via* inhibition of HMGA2 in lung cancer cells. Paclitaxel plus PD-L1 blockade treatments promoted CD8^+^ TILs recruitment due to CXCL10/CXCL11 upregulation (Liu J. et al., 2022). This study suggested that chemotherapy plus ICIs are effective in NSCLC patients with KRAS-G12D mutation (Liu C. et al., 2022).

### The efficacy of immunotherapy in EGFR-mutant NSCLC

EGFR-mutant lung cancer patients exhibit therapy resistance (Passaro et al., 2021; Girard, 2022). Activation of EGFR has been reported to establish an immunosuppressive TME in NSCLC cells, including promotion of suppressive TAMs, Tregs, blockade of T cell infiltration and cytotoxicity, and induction of inhibitory cytokines, which impair the immunotherapy (Lin et al., 2019). About 50% of NSCLC patients with EGFR mutations acquired EGFR-tyrosine kinase inhibitor (TKI) resistance. EGFR pathway has been reported to regulate PD-L1 in NSCLC (Hsu et al., 2019). EGFR-TKI resistance upregulated PD-L1 expression and caused immune escape in lung cancer *via* activation of phosphatidylinositol-3 kinase (PI3K), mitogen-activated protein kinase (MAPK) and NF-kappa B (NF-κB) pathways (Peng et al., 2019). One study found that hypoxia-inducible factor 1α (HIF-1α) and NF-κB are critical to modulate the expression of PD-L1 in EGFR-mutant NSCLC cells (Guo et al., 2019). Another group suggested that TGF-β/Smad pathway participated in PD-L1-mediated EGFR-TKIs resistance in NSCLC with EGFR mutations (Zhang et al., 2019). Overexpression of PD-L1 increased gefitinib resistance in EGFR-mutant NSCLC cells, while depletion of PD-L1 reduced gefitinib resistance (Zhang et al., 2019). Activation of OPN/integrin αVβ3/FAK pathway is important for regulation of EGFR-TKI resistance in NSCLC with EGFR mutations (Fu et al., 2020). PD-L1 expression is correlated with TKIs response and prognosis in lung cancer patients with EGFR mutations (Lin et al., 2015).

Immunoglobulin-like transcript 4 (ILT4) belongs to the immunoglobulin superfamily and often expressed in myeloids, which can promote the proliferation, migration and invasion in human cancers. ILT4 induced immunosuppressive T cell infiltration and led to poor prognosis in lung cancer. ILT4 stimulated T cell senescence and reduced tumor immunity in the TME in human cancer (Gao et al., 2021; Yang et al., 2021). Moreover, ILT4 acts as a useful checkpoint molecule for immunotherapy (Gao et al., 2018). One group showed that ILT4 expression can be elevated after EGFR activation in NSCLC cells, which was mediated by activated ERK and AKT cellular signaling pathways (Chen K. et al., 2021). Moreover, ILT4 increased recruitment of TAMs and M2-like polarization in NSCLC cells with EGFR activation, leading to impairing proliferation and cytotoxicity of T cells (Chen X. et al., 2021). Furthermore, inhibition of ILT4 promoted the efficacy of PD-L1 inhibitors and abrogated TAMs- and T cell-involved immunosuppression in NSCLC cells with EGFR activation. *In vivo* study showed that knockdown of ILT4 and PD-L1 blockade synergistically retarded mouse tumor growth and inhibited immune escape (Chen K. et al., 2021). Animal study data further showed that inhibition of ILT4 alone repressed tumor progression and immune evasion in EGFR mutant NSCLC. This work implied that inhibition of ILT4 increased the efficacy of immunotherapy in EGFR-mutant NSCLC (Chen X. et al., 2021). One retrospective study determined the association between PD-L1, TILs and immunotherapy response in uncommon EGFR-mutant NSCLC patients (Chen et al., 2020). Among 600 NSCLC cases with EGFR mutations, 49 cases were borne with uncommon alterations, such as Ex20, L861Q, S7681, G719X. Uncommon EGFR-mutant NSCLC patients had a high PD-L1 expression and CD8^+^ TILs and displayed a favorable response to anti-PD-1 therapy (Chen et al., 2020). Therefore, like in common EGFR-mutant NSCLC patients, combination of CD8^+^ TILs and PD-L1 level in TME can determine the anti-PD-1/PD-L1 therapy efficacy for NSCLC patients with uncommon EGFR mutations (Chen et al., 2020). Anti-CD73 in combination with anti-PD-L1 therapy enhanced T cell response *via* upregulation of the number of CD8^+^ T cells and promotion of TNF-α and IFN-γ production in EGFR-mutant NSCLC, leading to inhibition of tumor growth (Tu et al., 2022).

ERBB-family genetic alterations and KRAS mutations regulated response to anti-PD-1 inhibitors in NSCLC with metastasis (Cinausero et al., 2019). NSCLC patients with KRAS mutations had a better anti-PD-1 therapy efficacy and a longer PFS and OS. NSCLC patients with EGFR mutation, ERBB2 mutation and ERBB4 mutations had a worse response to anti-PD-1 therapy (Cinausero et al., 2019). STK11/LKB1 mutations were linked to resistance of PD-1 blockade in KRAS-mutant lung cancer (Skoulidis et al., 2018). Biton et al. (2018) also reported that TP53, STK11, and EGFR mutations represented the anti-PD-1 treatment efficacy in lung adenocarcinoma. NSCLC patients with STK11 mutation displayed chemotherapy resistance, while co-mutations with KRAS or TP53 modulated TIME of STK11-mutant NSCLC tumors and affected immunotherapy response (Malhotra et al., 2022). Additionally, NSCLC patients with EGFR/HER2 exon 20 insertions had a higher expression of PD-L1 and exhibited the sensitive to anti-PD-1/PD-L1 therapy (Chen X. et al., 2021).

### The efficacy of immunotherapy in ALK-rearranged NSCLC

ALK-rearranged tumors exhibited more resting memory CD4^+^ T cells and less activated memory CD4^+^ T cells and CD8^+^ T cells (Jin et al., 2020). Anti-PD-1/PD-L1 therapy is useful for the treatment of ALK-translocated NSCLC patients (Bylicki et al., 2017). ALK positivity and EGFR mutations have been reported to be adverse predictors for NSCLC patients (Bahnassy et al., 2022). A retrospective analysis showed that ALK rearrangement and EGFR mutations were involved in poor response to blockade of PD-1 pathway in NSCLC (Gainor et al., 2016). This could be due to low rates of PD-L1 expression and CD8^+^ TILS in the TME in NSCLC patients (Gainor et al., 2016). Similarly, PD-L1 expression and CD8^+^ T cells infiltration have a clinical relationship in lung cancer patients with ALK-rearranged and EGFR-mutated tumors (Liu et al., 2018). Lung cancer patients with ALK-rearrangement or EGFR mutations had lowest proportion of PD-L1+/CD8+ tumors and the shortest overall survival. Lung cancer patients with ALK-rearrangement or EGFR mutations showed a poor response to ICIs (Liu et al., 2018). Strikingly, PD-L1 expression and CD8 expression are biomarkers for prediction of prognosis with poor prognosis in patients with EGFR mutations or ALK rearrangement (Liu et al., 2018). Interestingly, a retrospectively study indicated that cytotoxic chemotherapy affected the TIME in NSCLC patients with wild type of ALK and EGFR (Sakai et al., 2019). Platinum-based adjuvant chemotherapy modulated PD-L1 expression, CD8^+^ TIL density and tumor mutation burden (TMB) in NSCLC patients (Sakai et al., 2019).

### The efficacy of immunotherapy in ROS1-rearrangement NSCLC

One research group reported that expression of ROS1 and ROS1-rearrangement was observed in 18.57% and 15.71% of the 70 NSCLC patients, respectively (Bahnassy et al., 2022). ROS1 expression was not correlated with PD-1, PD-L1, survival and therapy response (Bahnassy et al., 2022). Another research simultaneous genotypic screening of three gene mutations, including ROS1, ALK and EGFR, to measure the prevalence and clinicopathologic features of ROS1 mutations and immunotherapy efficacy in NSCLC patients (Lee et al., 2019). This study found that among 407 NSCLC cause, there were 14 ROS1 and 19 ALK rearrangements and 106 EGFR mutations. Among 130 NSCLC tumors, 29 samples had high expression of PD-L1. Among 14 cases with ROS1 mutations, 12 samples exhibited PD-L1 expression and 5 cases displayed high expression level of PD-L1 expression (Lee et al., 2019). This work indicated that ROS1 rearrangement was overlapped with high expression of PD-L1 in NSCLC patients (Lee et al., 2019). Similarly, the correlation among oncogenic mutations in ROS1, ALK, EGFR and PD-L1 had been reported in lung adenocarcinoma (Rangachari et al., 2017). This retrospective work explored 71 cases of lung cancer and found that 29.6% cases had a PD-L1 TPS of high than 50%. Of 19 cases with ALK, ROS1, or EGFR mutations, 18 cases had a PD-L1 TPS less than 50%. Moreover, lung cancer with a PD-L1 TPS of high than 50% was correlated with smoking status (Rangachari et al., 2017). In addition, it has been compared with ALK, ROS1, EGFR, and PD-L1 between cytological tumors and surgical tumors in NSCLC to explore the adequacy of PD-L1 expression by a retrospective study (Ekin et al., 2021). Among 220 NSCLC cases, there were 64 small histology biopsies, 90 surgical biopsies and 66 cytology samples. However, there was no difference between two types of samples (154 histological plus surgical and 66 cytology samples) in cellular adequacy for EGFR, ROS1, ALK, and PD-L1. There was no change in the expression positivity rates for these four biomarkers between two types of samples (Ekin et al., 2021). ROS1-rearranged lung adenocarcinoma patient had active TME and increased plasma inflammatory factors when the patient received immune therapy and ceritinib chemotherapy. PD-L1 expression was elevated in tumor samples during treatment, suggesting that the patient obtained a limited benefit from combination therapies (Yue et al., 2021).

### The efficacy of immunotherapy in TP53-mutant NSCLC

An immunohistochemical work illustrated that PD-L1 expression was associated with poor overall survival and PFS in NSCLC patients. CD8^+^ TILs were correlated with therapy response and a good PFS and overall survival. P53 expression was observed in most of NSCLC samples, but was not correlated with PD-L1 expression (Rashed et al., 2017). Serra et al. (2018) found that RAS/TP53 mutations were associated with PD-L1 expression in lung adenocarcinoma. Moreover, Dong et al. (2017) uncovered that TP53 mutation and KRAS mutation can predict the response to anti-PD-1 immunotherapy in lung adenocarcinoma. Zhang L. et al. (2021) reported that 219 cases from 350 NSCLC patients harbored TP53 mutations. Coexisting TP53 and ZFHX3 mutations were correlated with prognosis, indicating that TP53 and ZFHX3 mutations could be prognostic factors for late-stage NSCLC cases undergoing anti-PD-1/PD-L1 therapy. Another study also clarified that TP53 mutations were associated with response to ICIs treatment and a longer survival in advanced NSCLC patients (Assoun et al., 2019). Notably, NSCLC patients with TP53 plus KEAP1 mutations had a better PFS after treatment with PD-1/PD-L1 monotherapy (Wang S. et al., 2022). Strikingly, the TP53-missense mutation patients displayed enriched IFN-γ signatures and TME composition compared with TP53 wild-type patients (Sun et al., 2020). TP53 non-sense mutation patients exhibited promotion of suppressor immune cells, such as M2 Macrophage and Neutrophils. Upregulation of TMB and neoantigen levels were observed in both TP53 non-sense and missense mutations. TP53 missense was linked to better benefit of anti-PD-1/PD-L1 therapy (Sun et al., 2020).

### The efficacy of immunotherapy in PTCH1-mutant NSCLC

Patched 1 (PTCH1) is one component of hedgehog pathway, which has been correlated with tumor malignancies (Sigafoos et al., 2021). In NSCLC patients, PTCH1 was underexpressed in the tumor specimens compared with normal lung samples (Herreros-Pomares et al., 2022). NSCLC patients with overexpression of PTCH1 displayed a better outcome (Herreros-Pomares et al., 2022). Moreover, PTCH1 expression was found to be correlated with NSCLC development (Barbirou et al., 2022). One circulating tumor cell NGS assay in early-stage lung cancer patients showed that more than 50% of lung cancer patients presented four common mutations, including Notch1, EGFR, IGF2, and PTCH1 (Wan et al., 2021). Genetic mutation analysis demonstrated that 147 mutant genes were discovered in small cell lung cancer patients, including TP53, RB1, KMT2D, PTCH1, APC, LRRK2, ARID2, and BRCA1 (Jin et al., 2021). In addition, elevated mutations of six genes were linked to advanced clinical stages II and III, such as SETD2, WT1, EPHA3, ACVR1B, NOTCH1 and KDM6A (Jin et al., 2021). Similarly, TP53 and RB1 mutations are two most frequently mutations in SCLC, while FGFR1, KIT, PTCH1, RICTOR, and RET mutations are low-frequency mutations (Dowlati et al., 2016). One retrospective study used the data from 180 lung squamous cell carcinoma and reported that patched receptor 1 (PTCH1) gene mutation was linked to CD8^+^ TILs density (Cheng et al., 2022). CD8^+^ TILs and high expression of PD-L1 were correlated with better disease-free survival in lung squamous cell carcinoma patients (Cheng et al., 2022). Serial sequencing of circulating tumor DNA (ctDNA) showed that PTCH1 mutation and β2 microglobulin (B2M) mutations were observed in NSCLC patients with anti-PD-1 treatment. Moreover, PTCH1 and B2M mutations were associated with distant metastasis in NSCLC patients (Li et al., 2019).

### The efficacy of immunotherapy in ZFHX3-mutant NSCLC

ZFHX3 was reported to suppress alpha-fetoprotein expression. ZFHX3 mRNA expression in tumor tissues was linked to overall survival rate in 140 NSCLC patients. Low expression of ZFHX3 in NSCLC patients was associated with LNM and poor prognosis (Minamiya et al., 2012). Song et al. (2021) reported genomic profiles and TIME of lung cancer with brain metastasis. High-frequent ZFHX3 was found in 40% lung tumors and 28% brain tumors. A majority of lung cancer patients had lesions-shared mutations, such as EGFR mutation. Zhou et al. (2020) reported that 19% ZFHX3 mutation frequency was identified in lung cancer patients by next-generation sequencing. Another study also identified that the mutation of ZFHX3 in NSCLC patients could have benefit from ICIs treatment (Principe, 2022). ZFHX3 was identified as a genomic mutation for prediction of immunotherapy in NSCLC patients (Wang Z. et al., 2022). ZFHX3 mutation in NSCLC patients was correlated with TILs, immune-related gene expression and tumor mutation burden. ZFHX3 mutation was also linked to longer overall survival of NSCLC patients after treatment with ICIs (Zhang J. et al., 2021).

### The efficacy of immunotherapy in PAK7-mutant NSCLC

Evidence has shown that p21-activated kinase (PAK7) regulates carcinogenesis in a variety of malignancies (Gu et al., 2013; Han et al., 2014; Quan et al., 2020; Wang et al., 2020). Suppression of PAK7 increased radio-sensitivity in hepatocellular carcinoma (HCC) (Gu and Kong, 2021). Depletion of PAK7 by shRNA transfection induced apoptosis and G2/M phase arrest, decreased clone formation and elevated γ-H2AX expression in HCC cells (Gu and Kong, 2021). PAK7 expression was upregulated in breast tumor samples and associated with differentiation and TNM stage in breast cancer patients. PAK7 activated Wnt/*β*-Catenin pathway and caused promotion of proliferation and migration as well as inhibition of apoptosis in breast cancer (Li et al., 2018). In esophageal squamous cell cancers (ESCC), high expression of PAK7 was correlated with LNM (He et al., 2016). Moreover, PAK7 was regulated by Aurora-A *via* binding with E2F1 in ESCC cells. PAK7 induced cisplatin resistance of ESCC with Aurora-A overexpression (He et al., 2016). One group revealed that PAK7 could be related to gemcitabine resistance in NSCLC cells (Zhang et al., 2013). PAK7 mutations were found to be associated with tumor mutation burden, neoantigen load, copy number variation, CD8^+^ TILs, mutation rate in the DDR-related pathways, suggesting that PAK7 mutations could be a helpful biomarker for prediction of the immunotherapy efficacy in NSCLC patients (Zeng et al., 2022).

### The efficacy of immunotherapy in UBE3A-mutant NSCLC

UBE3A, also known as E6AP, acts as an E3 ligase and critically involves in carcinogenesis (Owais et al., 2020; Zheng et al., 2021). For example, UBE3A promoted tumor progression *via* disruption of ZNF185 in ESCC (Zheng et al., 2021). UBE3A targeted SIRT6 and regulated liver tumorigenesis, which was dependent on ANXA2 (Kohli et al., 2018). Downregulation of E6AP led to decreased expression of p15, p16 and p19 in NSCLC. E6AP represses the expression of CDC6 *via* inhibiting its E2F1 transcription (Gamell et al., 2017). UBE3A deletion promoted the immunotherapy efficacy in NSCLC patients (Zhang L. X. et al., 2022). NSCLC patients with UBE3A deletion had higher TILs and higher expression of immune checkpoint biomarkers (Zhang N. et al., 2022).

### The efficacy of immunotherapy in TNF-α-mutant NSCLC

A mutated human tumor necrosis factor alpha (TNF-α) has been reported to improve the therapeutic index in the mouse fibroblast cell line L929 and mice (Yan et al., 2006). Similarly, TNF-α mutant was also found to promote cytotoxicity and receptor binding affinity (Shin et al., 1998). Pharmacokinetics of the recombinant mutated human TNF-α (rmhTNF-α) displayed that rmhTNF-α has a low systemic toxicity and high anticancer ability (Li et al., 2010). Phase II multicenter, randomized, double-blind trial showed that rmhTNF-α plus chemotherapies displayed higher response rate compared with chemotherapy alone group in multiple types of cancers. 11.39% patients had a response in the chemotherapy alone, while 27.47% patients had a response in the chemotherapy plus rmhTNF-α treatment. In lung cancer patients, the combination treatment caused 48.89% patients a response (Li et al., 2012). Moreover, a randomized phase III trial in stage IIIB/IV NSCLC patients showed that rmhTNF-α potentiated the efficacy of chemotherapy in advanced NSCLC patients (Ma et al., 2015). TNF-α alternation was uncovered for prediction of survival of ICIs in NSCLC patients. TNF-α mutations were linked to prolonged overall survival in NSCLC patients undergoing immunotherapy (Lin et al., 2021). TNF-α mutations were also related with TMB, DDR mutations and neoantigen load, and infiltrating immune cells (Lin et al., 2021).

### The efficacy of immunotherapy in NOTCH-mutant NSCLC

Notch signaling pathway is critically involved in tumorigenesis, which consists of four receptors, Notch1, Notch2, Notch 3, Notch4, and several ligands, such as delta-like proteins (DLL1, DLL3, DLL4), Jagged-1 and Jagged-2 (Gao et al., 2020; Majumder et al., 2021). In general, 20%–25% of SCLC patients exhibited Notch mutations with loss-of-function (LOF). Notch can act as a tumor suppressor in SCLC and also enhance non-neuroendocrine plasticity to facilitate tumor growth in SCLC (Hong et al., 2022). Mice with genetic loss of Nocth1 or Nocth2 facilitated SCLC tumorigenesis and formed non-neuroendocrine populations *via* regulation of RUNX2/REST pathway and STING (stimulator of interferon genes) (Hong et al., 2022). Li Y. et al. (2022) reported that Notch pathway was correlated with TIME in SCLC. Notch1 gene mutation was negatively linked to PD-L1 expression in SCLC patients. Higher expression of DLL3 was found in SCLC patients and associated with PD-L1 levels. Hence, SCLC patients with positive DLL3 expression and Notch1 wild type had PD-L1 overexpression, which could be likely to have good immunotherapy efficacy. Notch2 mutation was a prognostic factor in NSCLC patients and could be provide a new treatment option for patients without EGFR mutations (Niu et al., 2021).

The high-mutated NOTCH pathway could act as a biomarker for predicting the prognosis of ICIs-treated NSCLC patients because NSCLC patients with high-mutated NOTCH pathway had a better PFS and OS (Li et al., 2021). Zhang et al. (2020) also identified that Notch mutation acted as a new predictor for efficacious immunotherapy in NSCLC patients. Notch1/2/3 mutation had a correlation with better ICI treatment outcomes, including PFS and overall survival due to regulation of transcription of genes that were related to immune activation and DNA damage response (Zhang et al., 2020). Notch4 mutation was also a potential response biomarker for ICIs therapy in several cancer types, including NSCLC (Long et al., 2021). Cancer patients with Notch4 mutation displayed better responses for ICI therapy, including ORR, DCB, PFS and overall survival. Notch4 mutation was linked to increased immunogenicity, high TMB, anticancer immunity and activation of the antigen-processing machinery (Long et al., 2021).

### The efficacy of immunotherapy in LRP1B-mutant NSCLC

LRP1B has been reported to be frequently mutated in numerous types of cancers, including lung cancer (Principe et al., 2021). The bioinformatics analysis showed that LRP1B mutation was linked to age and MUC16 and TP53 mutation status in gastric cancer patients (Hu et al., 2021). The next-generation sequencing (NGS) data showed that 13.98% of NSCLC patients had LRP1B mutation (Xu et al., 2023). LRP1B mutation was correlated with high TMB in NSCLC. Moreover, NSCLC patients with LRP1B mutation had a high infiltrating levels of immune cells and immune molecules. Additionally, LRP1B mutations were linked to several pathways in the immune system, including cell cycle, Notch, mTOR and insulin pathways (Xu et al., 2023). LRP1B mutation was associated with TMB and outcomes in NSCLC patients with immunotherapy (Chen et al., 2019). LRP1B mutation was correlated with a better survival in NSCLC patients. Moreover, LRP1B mutations was also associated with immunocytes and enriched pathways, such as cell cycle mitotic, antigen processing and presentation pathways (Chen et al., 2019). Another group reported that LRP1B mutation was correlated with better outcomes to ICIs in combination with chemotherapy in NSCLC patients (Zhou J. et al., 2022). Hence, LRP1B mutations could be critical in promoting immunotherapy and might be a biomarker for judgement of treatment responsiveness.

### The efficacy of immunotherapy in FBXW7-mutant NSCLC

FBXW7, one of F-box proteins, has been identified to regulate carcinogenesis and progression (Wang et al., 2014; Yan et al., 2020; Liu F. et al., 2022). FBXW7 mutation caused drug resistance *via* targeting several downstream substrates for ubiquitination and degradation, including Mcl-1, mTOR, snail and CCDC6 in NSCLC (Peng and Chen, 2019). Several compounds displayed an effective treatment efficacy in NSCLC patients with FBXW7 mutation, such as rabdosia, MS-275 and rapamycin (Peng and Chen, 2019). By analysis of TCGA data, 30.9% of lung adenocarcinoma presents FBXW7 deletion, and 63.5% of lung squamous cell carcinoma exhibited FBXW7 deletion. FBXW7 deletion led to lung oncogenesis and contributed to gefitinib resistance (Xiao et al., 2018). One study revealed that 5.6% of NSCLC patients (7 cases) had FBXW7 truncating mutations in 125 NSCLC cases. In these seven patients with FBXW7 mutation after they obtained immunotherapy, four cases presented partial response, two cases showed stable disease, and one case displayed progressive disease (Liu J. et al., 2022). FBXW7-mutant NSCLC patients had 13 months for median progression-free survival (PFS), while FBXW7 wild type patients had 4 months for PFS. FBXW7-mutant patients had a higher TMB and the activation of T cells. Moreover, FBXW7 mutation was linked to upregulation of CD8^+^ T cell infiltration and M1 macrophages. FBXW7 gene mutation could predict the prognosis of immunotherapy in patients with NSCLC (Liu C. et al., 2022).

### LncRNAs and circRNAs regulate TME and immunotherapy in lung cancer

Besides these gene mutations, non-coding RNAs have been reported to involve in regulating TME and immunotherapy in lung cancer. Non-coding RNAs have been reported to involved in human cancer development and progression (Ghafouri-Fard et al., 2020; Yan and Bu, 2021; Chen T. et al., 2022; Liu and Shang, 2022). Evidence has shown that lncRNAs play an essential role in NSCLC initiation, development and progression (Osielska and Jagodzinski, 2018; Wang et al., 2018; Hu et al., 2022). Moreover, non-coding RNAs are critically involved in cancer drug resistance in human cancers (Jiang et al., 2020; Zhou X. et al., 2022; Xie et al., 2022). LncRNAs and exosomal lncRNAs regulate tumor progression, drug sensitivity and TME remodeling in lung cancer (Entezari et al., 2022). The role of lncRNAs in the regulation of PD-1 and PD-L1 pathways and TME in cancer immunotherapy has been discussed (Jiang W. et al., 2021; Dai et al., 2021; Zhang P. et al., 2022). For example, lncRNA C5orf64 was characterized as a potential indicator for TME and mutation pattern remodeling in lung cancer (Pang et al., 2021). LncRNA C5orf64 expression was positively associated with neutrophils, monocytes, M2 macrophages and eosinophils, and negatively linked to Tregs and plasma cells (Pang et al., 2021). High expression of C5orf64 was linked to upregulation of PD-1, PD-L1 and CTLA-4 expression. Interestingly, lung adenocarcinoma patients with high expression of C5orf64 had a low frequency of TP53 mutation (Pang et al., 2021). Together, lncRNA C5orf64 could be a useful indicator for TME modulation and immunotherapy in lung cancer. Jiang Y. et al. (2021) found that cancer-associated fibroblasts (CAFs)-derived exosomes can regulate lncRNA OIP5-AS1 and modulate miR-142-5p and control the expression of PD-L1, leading to promotion of lung cancer progression. Moreover, N6-methyladenosin (m6A) related lncRNA signatures with TME have been defined to predict the immunotherapy in lung cancer (Weng et al., 2021; Zhao et al., 2021; Zhang W. et al., 2022; Yan et al., 2022). Recently, the circular RNA circHMGB2 was uncovered to promote immunosuppression and resistance to anti-PD-1 therapy *via* targeting miR-181a-5p and upregulating CARM1 in lung cancer, suggesting that circHMGB2 reshaped the TME and governed immunotherapy in lung cancer (Zhang L. X. et al., 2022).

## Conclusion and perspectives

**FIGURE 2:**
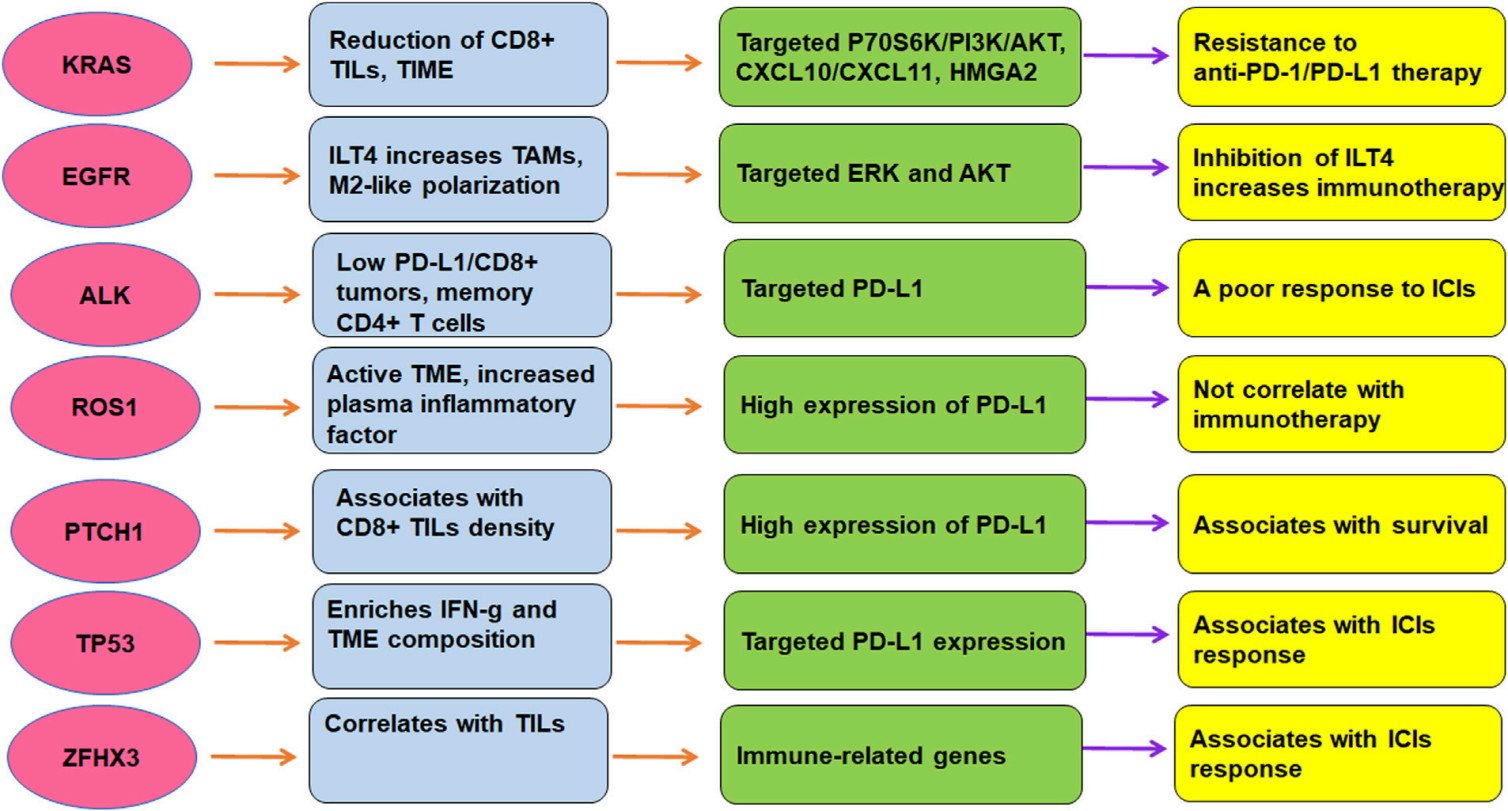
The role of gene mutations in regulation of TME and immunotherapy in lung cancer. The efficacy of immunotherapy was regulated by driven gene mutations in lung cancer, including KRAS, TP53, EGFR, ALK, ROS1, ZFHX3, and PTCH1.

TME is critically involved in immunotherapy in lung cancer. The efficacy of immunotherapy was regulated by driven gene mutations in lung cancer, including KRAS, TP53, EGFR, ALK, ROS1, ZFHX3, and PTCH1 (Figure 2). Targeting TME could abolish immune resistance of anti-PD-1/PD-L1 treatment in lung cancer. It has been suggested that PD-1/PD-L1 blockade should be combined with other therapy such as chemotherapy to improve the anticancer efficacy in human cancer (Wu et al., 2022).

Several issues need to be clarified regarding the TME and immunotherapy in NSCLC. For example, several reports showed that mRNA vaccine could be applied for cancer treatment *via* regulation of TME (Zhong et al., 2021; Zhao W. et al., 2022; Huang et al., 2022). Proteomics, genomics, and metabolomics might be good approaches to explore the mechanism of gene mutation-driven lung cancer and TME (Zhou et al., 2019; Bourbonne et al., 2022). Recently, several studies used the single-cell profiling of lung cancer to determine the TME and immunotherapy (Maynard et al., 2020; Wu et al., 2021; Hui et al., 2022). Hui et al. (2022) reported single-cell profiling of immune cells after chemotherapy and pembrolizumab in advanced NSCLC. This study found the synergistical increase of CD4^+^ cells and B cells were positively correlated with chemoimmunotherapy. Moreover, this work identified several positive outcomes, such as promotion of TNFRSF4+ Tregs, LAMP3+ DCs, intratumoral CD4^+^ T clones and CD8^+^ T clones (Hui et al., 2022). In addition, single-cell RNA sequencing was used to evaluate therapy-induced evolution in lung cancer patients, including TN (patients before initiating systemic therapy, TKI naive), RD (residual disease) and PD (on-therapy progressive disease) (Maynard et al., 2020). Transcriptional differences between RD and TN tumor cells suggested cell-state-specific programs, while transcriptional differences between PD and TN tumor cells indicated that immune modulation and invasion are critical for cancer progression. RD patients displayed active T-lymphocytes and reduced macrophages, while PD patients displayed immunosuppressive cell states (Maynard et al., 2020). Wu et al. also reported single-cell profiling of tumor heterogeneity and TME in advanced NSCLC. This work identified not only common cell types but also rare cell types in tumors including T helper 17 cells and follicular dendritic cells. Different NSCLC patients exhibited larger heterogeneity in chromosomal structure, intercellular signaling network and cellular composition and so on (194). Further investigations are necessary to determine the underlying molecular mechanisms of TME in regulation of immunotherapy resistance. In addition, besides TME, immunotherapy resistance could be caused by other factors in lung cancer, which should be explored in the future.

## Abbreviations

ALK, anaplastic lymphoma kinase; BRAF, v-Raf murine sarcoma viral oncogene homolog B; CNV, copy number variation; CTLA-4, cytotoxic T-lymphocyte antigen 4; DDR, DNA damage response and repair; EGFR, epidermal growth factor receptor; FBXW7, F-box and WD-40 domain protein 7; ICIs, immune checkpoint inhibitors; KRAS, Kirsten rat sarcoma; LRP1B, low-density lipoprotein receptor-related protein 1B; mTOR, mammalian target of rapamycin; NSCLC, non-small cell lung carcinoma; OS, overall survival; PD-1, programmed cell death-1; PD-L1, programmed cell death ligand-1; PFS, progression-free survival; ROS1, receptor tyrosine kinase c-ros oncogene 1; TMB, tumor mutational burden; TME, tumor microenvironment; TIME, tumor immune microenvironment; TNFα, tumor necrosis factor α; ZFHX3, zinc finger homeobox 3.
